# Assessing the performance of European-derived cardiometabolic polygenic risk scores in South-Asians and their interplay with family history

**DOI:** 10.1186/s12920-023-01598-5

**Published:** 2023-07-12

**Authors:** Emadeldin Hassanin, Carlo Maj, Hannah Klinkhammer, Peter Krawitz, Patrick May, Dheeraj Reddy Bobbili

**Affiliations:** 1 Luxembourg Centre for Systems Biomedicine, University of Luxembourg, 6 avenue du Swing, Belvaux, L-4367 Luxembourg; 2 Institute for Genomic Statistics and Bioinformatics, University of Bonn, Bonn, Germany; 3 Centre for Human Genetics, University of Marburg, Marburg, Germany; 4 Medical Faculty, Institute for Medical Biometry, Informatics and Epidemiology, University Bonn, Bonn, Germany; 5 Wellytics Technologies Pvt Ltd, Hyderabad, India

**Keywords:** Type 2 diabetes, Family History, South Asians, Polygenic risk, Coronary artery disease

## Abstract

**Background & aims:**

We aimed to assess the performance of European-derived polygenic risk scores (PRSs) for common metabolic diseases such as coronary artery disease (CAD), obesity, and type 2 diabetes (T2D) in the South Asian (SAS) individuals in the UK Biobank. Additionally, we studied the interaction between PRS and family history (FH) in the same population.

**Methods:**

To calculate the PRS, we used a previously published model derived from the EUR population and applied it to the individuals of SAS ancestry from the UKB study. Each PRS was adjusted according to an individual’s genotype location in the principal components (PC) space to derive an ancestry adjusted PRS (aPRS). We calculated the percentiles based on aPRS and stratified individuals into three aPRS categories: low, intermediate, and high. Considering the intermediate-aPRS percentile as a reference, we compared the low and high aPRS categories and generated the odds ratio (OR) estimates. Further, we measured the combined role of aPRS and first-degree family history (FH) in the SAS population.

**Results:**

The risk of developing severe obesity for SAS individuals was almost twofold higher for individuals with high aPRS than for those with intermediate aPRS, with an OR of 1.95 (95% CI = 1.71–2.23, P < 0.01). At the same time, the risk of severe obesity was lower in the low-aPRS group (OR = 0.60, CI = 0.53–0.67, P < 0.01). Results in the same direction were found in the EUR data, where the low-PRS group had an OR of 0.53 (95% CI = 0.51–0.56, P < 0.01) and the high-PRS group had an OR of 2.06 (95% CI = 2.00-2.12, P < 0.01). We observed similar results for CAD and T2D. Further, we show that SAS individuals with a familial history of CAD and T2D with high-aPRS are associated with a higher risk of these diseases, implying a greater genetic predisposition.

**Conclusion:**

Our findings suggest that CAD, obesity, and T2D GWAS summary statistics generated predominantly from the EUR population can be potentially used to derive aPRS in SAS individuals for risk stratification. With future GWAS recruiting more SAS participants and tailoring the PRSs towards SAS ancestry, the predictive power of PRS is likely to improve further.

**Supplementary Information:**

The online version contains supplementary material available at 10.1186/s12920-023-01598-5.

## Background

Several genome-wide association studies (GWAS) for more than 5000 traits in GWAS Catalog [1] have been conducted to date, and very few of the GWASs have had significant success translating into the clinical setting [2]. Hence, it is a significant milestone to translate GWAS findings to clinical settings, particularly for traits with high heritability. One of the drawbacks of the GWAS findings is that the identified genome-wide significant SNPs do not have such a large effect size in most cases. However, a current approach of combining those SNPs to a single score known as a polygenic risk score (PRS) has become popular to enhance the accuracy of predicting individuals at risk and has thus shifted the focus of the genetic community towards the use of GWAS findings again [3]. PRS can be a precious tool for risk stratification, particularly in identifying groups of people with extremely high or low genetic risk of developing a specific disease or trait. Moreover, based on our recent work and others, it has become clear that for certain traits, high PRS, along with rare disease-causing variants, can further increase the individuals’ risk of developing a disease compared to carriers without a high PRS [4–7].

Identifying the risk SNPs using GWAS requires a considerable sample size as even most disease-related SNPs have relatively small effect sizes. So far, most of the larger GWASs have been mainly conducted in individuals with European (EUR) ancestries. One of PRS limitations is that it may not be transferable between different ancestries [8]. Due to both potential gene-environment interactions and population structure the application of EUR GWAS derived PRS can be problematic in non-EUR populations as it often results in shifted PRS distribution [8]. This lack of portability of PRS is due to differences in linkage disequilibrium (LD), risk variants, effect sizes, and allele frequencies. Further, methods to genotype or impute the missing SNPs initially developed with samples of EUR ancestry can increase those differences [9]. The critical demand to advance polygenic prediction in non-European populations is not being met, as South Asian (SAS) groups, which form the largest ancestry group encompassing 23% of the world’s population [10], remain significantly underrepresented in existing GWAS studies. This underscores the imperative to substantially increase their participation in genetic research [11].

Despite ongoing efforts to increase global genetics research diversity, it will take still some time to attain sufficient GWAS sample sizes to identify population-specific risk SNPs. As mentioned earlier, PRS is a potent tool to identify the sub-populations at risk. However, this inability to use it across populations with different ancestries is an important research topic. Several studies were being performed to study the portability of EUR-derived PRSs into other ancestries and an SAS specific PRS has been developed for CAD using previously published GWAS statistics [10]. However, the majority of them had limited success [12–14]. The PRS derived from EUR performed poorly in African population [15] and similar results were observed in a Latino/Hispanic population for some traits [16]. While EUR-derived PRSs showed similar results for quantitative traits like blood count and anthropometric features, it performed poorly for blood pressure traits [17]. Others have shown a connection between PRS and genetic ancestry [12, 18]. In other words, the studies show that applying PRSs derived from the EUR population directly on other ancestries might not be ideal. However, few studies used an approach to developing an ancestry-adjusted PRS (aPRS) that is mainly derived from EUR and can be transferred to other ethnicities [19]. For example, a study showed a compromised solution where they found a minimal decrease in the prediction power of the PRS in SAS compared to EUR [20].

Recently, it has been shown that in breast cancer, the PRS derived from EURs with an ancestry correction performed well in the SAS population [14]. However, it is still unclear to what extent populations of EUR and SAS ancestry share the same genetic underpinnings of such cardiometabolic/lifestyle traits, and such an assessment is still missing. It is of utmost importance to perform this assessment because compared to other ethnicities, SAS individuals have an increased susceptibility to coronary artery disease (CAD), obesity, and type 2 diabetes (T2D) [21]. The interplay between PRS and family history (FH) in predicting the risk of various diseases has been a topic of interest in recent years [5, 22–24]. Although previous studies have examined the independent effects of FH and PRS, there is a lack of systematic research on the relative contributions and overlap of these factors across different types of familial risk in SAS.

Here, we systematically assessed the portability of the aPRS derived from EUR ancestry for obesity, CAD, and T2D to the SAS population and the interplay of FH and PRS in the same individuals. Hence, we used a published list of SNPs derived from the PGS catalog [25], then generated the aPRS and applied it to the EUR and SAS samples from the UK Biobank (UKB).

## Methods

### Data source

The UKB is a prospective study that collects data over a long period and recruits volunteers aged between 40 and 69, mostly from Scotland, Wales, and England, totaling over 500,000 individuals. All participants have provided written consent and collected data is available for research purposes. The UK Biobank Axiom Array was used to generate genotyping data, which included around 850,000 variants and the imputation of over 90 million variants [26].

### Study cohort

CAD and T2D diagnoses were based on self-reported illness codes and international Classification of Diseases (ICD)-10 and ICD-9 diagnosis codes, and Office of Population Censuses and Surveys (OPCS-4) procedure codes [3]. CAD was defined using ICD-10 codes (I21.X, I22.X, I23.X, I24.1, or I25.2), ICD-9 codes (410.X, 411.0, 412.X, or 429.79), OPCS-4 codes (K40.[1–4], K41.[1–4], K45.[1–5], K49.[1–2], K49.[8–9], K50.2, K75.[1–75.4], or K75.[8–0.9]), and self-reported illness codes 1075. T2D was defined using ICD-10 code E11.X, and self-reported illness codes 1223. Diagnosis of obesity was based on body mass index (BMI), with individuals having a BMI > 25 considered obese.

We then estimated genetic ancestries (EUR, and SAS) by projecting the samples in the 1000 genome project (1KGP) principal component (PC) spaces, while considering 1KGP superpopulations as a reference. The UKB conducted quality control for the genetic data, and the UKB processed files were used in downstream analysis. We analyzed individuals of EUR and SAS ancestry, and samples with discordant genotypic versus reported sex, sex chromosome aneuploidy, and high heterozygosity or missing genotype rates were considered as outliers (coded as “YES” in the fields 22,001, 22,019, and 22,027 respectively) and excluded from further analysis. We included only individuals who are unrelated up to the second degree, and from each pair of related individuals, one member was randomly retained (kinship coefficient > 0.0884, according to the UKB).

### Polygenic risk score analysis

PRSs were calculated using panels of SNPs identified in the previous studies [3, 27] and the effect sizes were downloaded from PGS catalog [21] using the ids PGS000027, PGS000013, PGS000014 for BMI, CAD and T2D respectively. PRSice-2 was used to generate the PRS, which account automatically for allele-flipping and removing ambiguous SNPs [28]. Strand-ambiguous SNPs are the ones with A/T or C/G alleles. Since many GWAS studies do not report the strand assignments, it is a standard practice in PRS calculations to exclude ambiguous SNPs. Since we already obtained the list of SNPs for the PRS calculation, we utilized the *‘–no clumping’* and *‘–no regress’* parameters along with the other default parameters, to bypass the time-consuming steps of regression and clumping. PRS values were standardized using the mean and standard deviation for the whole data.

### Adjustment of PRS

Based on an previously applied approach [5, 19] to reduce the variation in the PRS distribution due to genetic ancestry, we calculated an adjusted PRS (aPRS). A linear regression model was fitted using the PRS as the outcome variable and the first four PC derived from UKB as covariates (PRS ~ PC1 + PC2 + PC3 + PC4). A predicted PRS was calculated based on this model. Finally, the aPRS was calculated by subtracting the predicted PRS from the raw PRS and standardized using the mean and standard deviation.

### Statistical analysis

To investigate the association of aPRS and disease risk, we used logistic regressions with the occurrence of the disease as an outcome, i.e., separate logistic regressions for CAD, T2D, and obesity, respectively. All analyses were done for SAS and EUR populations separately.

First, we used aPRS as a continuous variable and adjusted the model for age, sex, and the first four PCs corresponding to the model

$$\begin{array}{l}logit\left( {P\left( {Y = 1} \right)} \right) = {\beta _0} + {\beta _{aPRS}}aPRS + {\beta _{sex}}sex\\+ {\beta _{age}}age + \sum\limits_{k = 1}^4 {{\beta _{P{C_k}}}} P{C_k}\end{array}$$

with $Y=1$ corresponding to the occurrence of the disease (CAD, T2D or obesity). Adjusted odds ratios (ORs) were calculated as $OR=\text{e}\text{x}\text{p}\left({\beta }_{aPRS}\right)$.

Second, we categorized the aPRS into three groups: low aPRS, intermediate aPRS, and high aPRS. We used the percentiles of the aPRS distribution in the SAS and EUR populations, respectively. SAS individuals were assigned to the “low” aPRS group if their aPRS fell below the 20th percentile (“< 20%”) of the aPRS distribution in the SAS population and to the “high” aPRS group if their aPRS fell above the 80th percentile (“> 80%”) of the aPRS distribution in the SAS population. The remaining SAS individuals were assigned to the “intermediate” aPRS group (“20%-80%”). The same was done for EUR individuals based on the aPRS distribution in the EUR population.

Then we replaced the continuous aPRS variable in the logistic regression by the aPRS group using the “intermediate” aPRS group as the reference category, i.e., we used the model

$$\begin{array}{l}logit\left( {P\left( {Y = 1} \right)} \right) = {\beta _0} + {\beta _{aPR{S_{low}}}}aPR{S_{low}}\\+ {\beta _{aPR{S_{high}}}}aPR{S_{high}} + {\beta _{sex}}sex\\+ {\beta _{age}}age + \sum\limits_{k = 1}^4 {{\beta _{P{C_k}}}} P{C_k}\end{array}$$

with $aPR{S}_{low}=1$, if the individual is in the low aPRS group and 0 otherwise (analogous for $aPR{S}_{high}$). Adjusted ORs for disease occurrence when being in the low or high aPRS group compared to the intermediate aPRS group were calculated as $OR=exp\left({\beta }_{aPR{S}_{low}}\right)$ and $OR=exp\left({\beta }_{aPR{S}_{high}}\right)$ respectively.

Finally, to determine the combined effect of aPRS and family history (FH), we reclassified the three aPRS groups into six groups based on FH status. FH was defined as positive (and encoded as $FH=pos$) or negative (encoded as *FH* = neg) whether the individual has FH of the corresponding disease in parents or siblings. For example, individuals with high aPRS and positive family history are encoded as aPRS_high_FH_pos_. We then fitted the logistic regression model

$$\begin{array}{l}logit\left( {P\left( {Y = 1} \right)} \right) = {\beta _0} + {\beta _{aPR{S_{low}}F{H_{pos}}}}aPR{S_{low}}F{H_{pos}}\\+ {\beta _{aPR{S_{low}}F{H_{neg}}}}aPR{S_{low}}F{H_{neg}}\\+ {\beta _{aPR{S_{int}}F{H_{pos}}}}aPR{S_{int}}F{H_{pos}}\\+ {\beta _{aPR{S_{high}}F{H_{neg}}}}aPR{S_{high}}F{H_{neg}}\\+ {\beta _{aPR{S_{high}}F{H_{pos}}}}aPR{S_{high}}F{H_{pos}}\\+ {\beta _{sex}}sex + {\beta _{age}}age + \sum\limits_{k = 1}^4 {{\beta _{P{C_k}}}} P{C_k}.\end{array}$$

The reference category is then given by individuals with intermediate aPRS and without positive FH. The adjusted OR for the occurrence of disease of individuals with, e.g., high aPRS and positive FH compared to the reference category is then estimated by $OR=exp\left({\beta }_{aPR{S}_{high}F{H}_{pos}}\right)$.

### Model performance

For assessing the performance of the different models, the area under the curve (AUC) was used. The R package pROC was used to compute the AUC with 95% confidence intervals (CIs), and AUC. We randomly divided the data into (75%) training and (25%) testing datasets. Logistic regression models were fitted on the training data set, and model prediction and AUC calculations were made using the testing data set by applying the corresponding models. Additionally, we measured the area under Precision-Recall (PR) curve (AUPRC) using the R package *PRROC* to address the challenge of imbalanced datasets. Since case-controls ratios for T2D, and CAD were substantially higher in the SAS than EUR samples, for additional validation of our models we performed down sampling for SAS population to achieve the same case-control ratios. While for obesity, case-control ratio was roughly the same between both populations.

### Survival analysis

To calculate the cumulative lifetime risk based on aPRS strata and FH status, a Cox proportional hazard model was used. Again, separate models were fitted for each phenotype (CAD, T2D, and obesity) respectively. The occurrence of the disease was considered as the event variable. At the same time, age served as the time scale, i.e., the age at diagnosis was considered as event time for observed cases and the age at the most recent visit for censored control. Adjusted survival curves were produced considering the aPRS group, age, sex, FH, and the first four ancestry PCs. We used the Schoenfeld individual test to test the proportional hazard assumption for each variable. R packages *survival* and *survminer* were used to perform Cox proportional hazard models and test the proportional hazard assumption, and R 4.2.2 was used for all statistical calculations.

## Results

### UK biobank dataset description

We identified a total of 24,156 CAD cases among individuals of EUR ancestry and 822 SAS cases, with a mean age of 61.51 and 58.71 years at recruitment, respectively. The remaining individuals were considered controls. For T2D, we identified 25,526 cases among EUR individuals and 1,718 cases among SAS individuals, with a mean age of 60.39 and 57.42 years, respectively. For obesity (BMI > 25), we identified 301,385 EUR and 5,690 SAS cases, with a mean age of 55.73 and 53.80 years, in EUR and SAS, respectively (Table 1).

**Table 1:** Characteristics of the participants by CAD, T2D, and Obesity diagnosis. Coronary artery disease (CAD), type 2 diabetes (T2D), European (EUR), South Asian (SAS)

	*CAD*				*T2D*				*Obesity*			
*Ethnicity*	**EUR**		**SAS**		**EUR**		**SAS**		**EUR**		**SAS**	
*Diagnosis*	Cases	Controls	Cases	Controls	Cases	Controls	Cases	Controls	Cases	Controls	Cases	Controls
*Participants, N*	24,156	428,610	822	7842	25,526	427,240	1718	6946	301,385	149,889	5690	2773
*Male, N * *(%)*	18,514 (76.64)	188,835 (44.06)	690 (83.94)	3959 (50.48)	15,756 (61.73)	191,593 (44.84)	1055 (61.41)	3594 (51.74)	154,908 (51.39)	51,703 (34.49)	2993 (52.60)	1493 (53.84)
*Female, N * *(%)*	5642 (23.36)	239,775 (55.94)	132 (16.06)	3883 (49.52)	9770 (38.27)	235,647 (55.16)	663 (38.59)	3352 (48.26)	146,477 (48.61)	98,186 (65.51)	2697 (47.40)	1280 (46.16)
*Age, mean (SD)*	61.51 (6.19)	56.53 (8.03)	58.71 (7.69)	53.05 (8.35)	60.39 (6.77)	56.58 (8.04)	57.42 (7.79)	52.64 (8.34)	55.73 (7.64)	56.81 (8.02)	53.8 (8.36)	53.1 (8.65)
*Family history of CAD, N (%)*	14,759 (61.1)	188,340 (43.94)	457 (55.6)	3193 (40.72)	13,131 (51.44)	189,968 (44.46)	754 (43.89)	2896 (41.69)	138,185 (45.84)	64,259 (42.87)	2411 (42.37)	1159 (41.79)
*Family history of T2D, N (%)*	5290 (21.9)	90,086 (21.02)	431 (52.43)	4144 (52.84)	10,120 (39.65)	85,256 (19.96)	1113 (64.78)	3462 (49.84)	68,794 (22.82)	26,267 (17.52)	3059 (53.76)	1430 (51.56)

In the SAS population, CAD cases were more common in individuals a positive FH of CAD than individuals without FH of CAD with OR 1.98 [1.70–2.31], P < 0.01. Moreover, T2D was diagnosed significantly more frequently in individuals with a positive FH of T2D than in individuals without FH of T2D (OR = 2.09 [1.86–2.34], P < 0.01).

### Ancestry correction and PRS distribution within the UKBB cohort

**Fig. 1:**
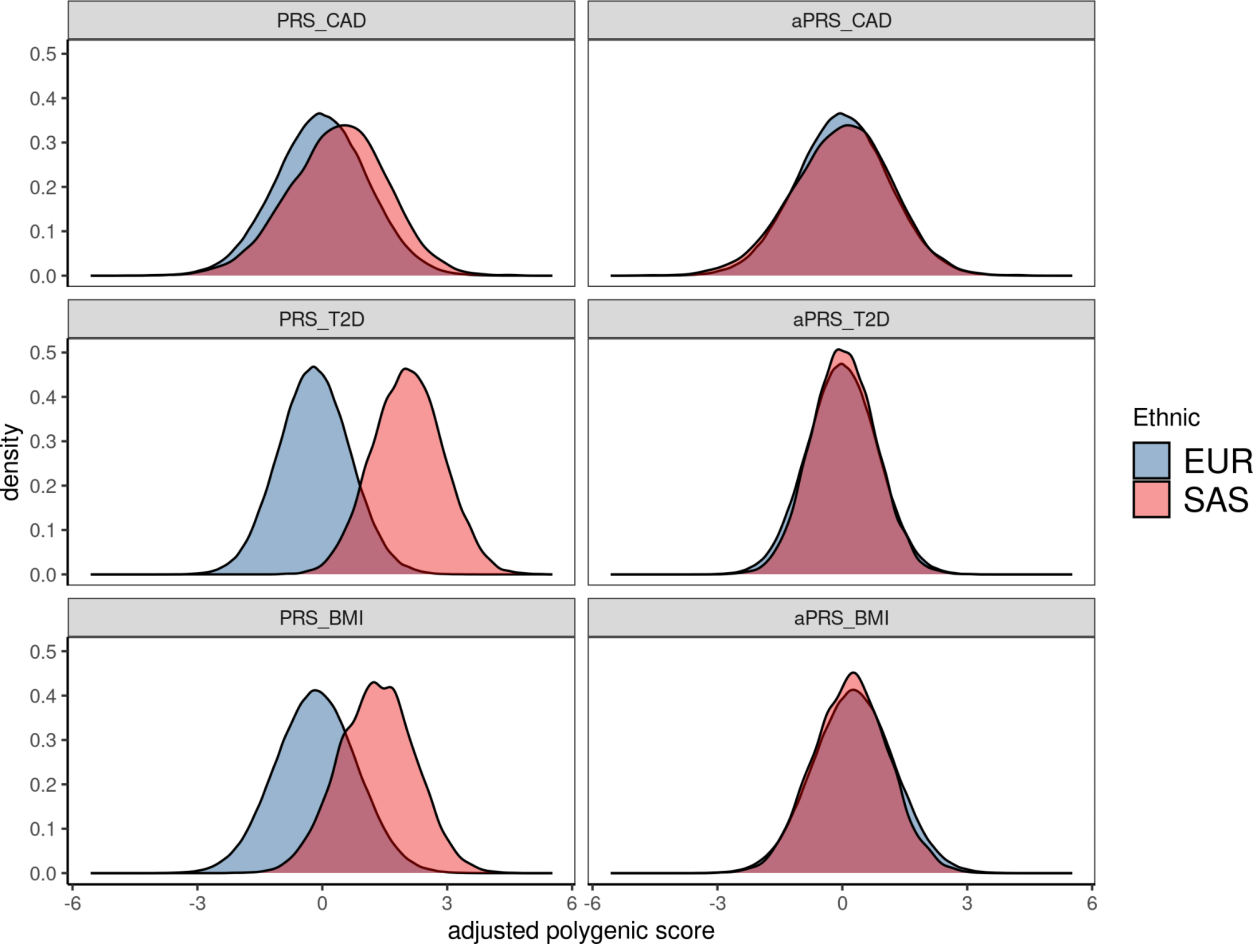
The distribution of PRSs before and after ancestry corrections across the various diseases. European (EUR), South Asian (SAS), coronary artery disease (CAD), type 2 diabetes (T2D), and adjusted polygenic risk scores (aPRS).

When studying individuals of a particular ancestry, it is crucial to apply ancestry correction using principal components (PCs) derived from the reference population. Figure 1 illustrates the effect of this step; while the PRS distributions are shifted horizontally for EUR and SAS populations, the ancestry correction ensures zero-centered aPRS distributions for each population. However, when using only PRS without ancestry correction, we observed a striking difference in the number of individuals assigned to high PRS (where high PRS was defined as an individual belonging to a PRS percentile > 80%). Specifically, there were significant variations between ethnic groups (EUR and SAS). In cases where matched reference controls are available, ancestry correction might not be necessary. However, due to the underrepresentation of SAS populations in current genetic studies, it is crucial to explore alternative approaches when ancestry-matched reference controls are not accessible. This will ensure more accurate and applicable results for diverse populations. For example, 18.5% of EUR samples (83,955/452,766) had a high PRS, while almost all SAS samples (96.2%, 8,331/8,664) showed a high PRS. However, applying aPRS reduced this variation. For instance, 20% of EUR samples (90,627/452,766) and 19.2% of SAS samples (1,659/8,664) had a high aPRS, leading to a more comparable distribution of PRS across ethnic groups. Similar results have been observed for CAD and obesity as well (Table 2). Our findings are in line with a previous study where they showed that ancestry correction is crucial to place an individual in the correct aPRS percentile for disease risk prediction [20].

**Table 2:** Comparison of the distribution of high (a) PRS (defined as PRS percentile > 80%). Coronary artery disease (CAD), type 2 diabetes (T2D), European (EUR), South Asian (SAS), Adjusted (a) polygenic risk scores (PRS)

	Ancestry correction	EUR samples with High PRS	SAS samples with High PRS
**T2D**	**PRS**	83,955 (18.5%)	8331 (96.2%)
	**adjusted PRS (aPRS)**	90,627 (20%)	1659 (19.2%)
**CAD**	**PRS**	89,438 (19.8%)	2,848 (32.9%)
	**adjusted PRS (aPRS)**	90,440 (20%)	1,846 (21.3%)
**Obesity**	**PRS**	85,853 (19%)	6433 (74.3%)
	**adjusted PRS (aPRS)**	90,794 (20.1%)	1492 (17.2%)

### Performance of aPRS on SAS individuals and association with disease development

**Fig. 2:**
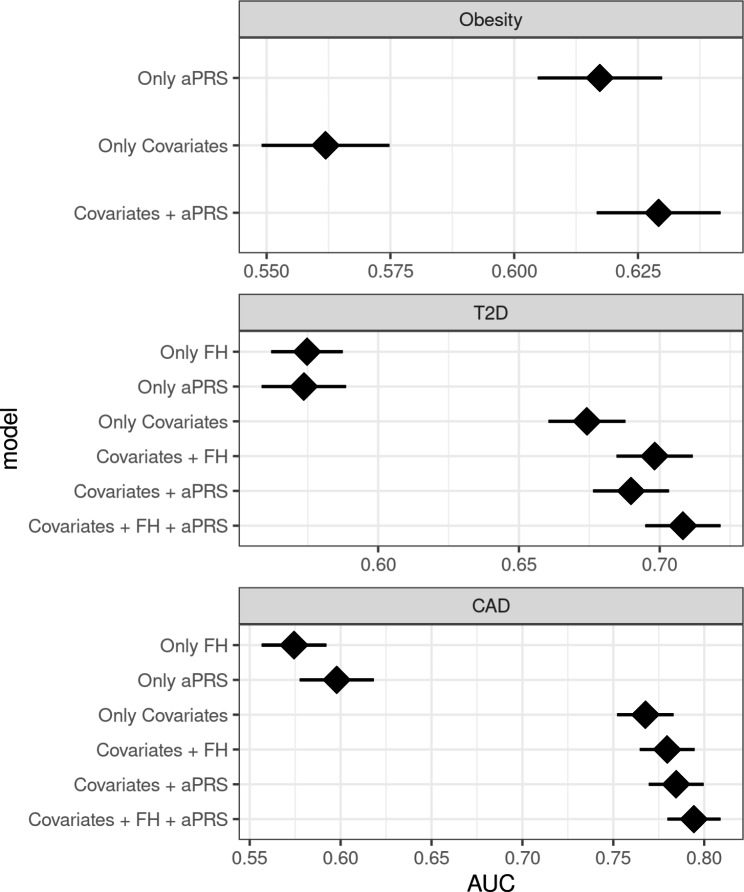
Comparison of different models and their corresponding AUCs among South-Asian (SAS) population. Ancestry adjusted PRS (aPRS), First degree family history (FH) and covariates (age, sex, first four principal components). European (EUR), coronary artery disease (CAD), type 2 diabetes (T2D).

Our analysis revealed that the models incorporating both adjusted polygenic risk scores (aPRS) and covariates have improved performance compared to the models based solely on covariates. This was evident from the higher AUC values for all three conditions - obesity, coronary artery disease (CAD), and type 2 diabetes (T2D) - when aPRS was included in covariates models. Specifically, for obesity, the AUC increased from 0.56 (95% CI, 0.55–0.57) to 0.63 (95% CI, 0.62–0.86); for CAD, it rose from 0.76 (95% CI, 0.75–0.78) to 0.79 (95% CI, 0.77–0.8); and for T2D, it increased from 0.67 (95% CI, 0.66–0.68) to 0.69 (95% CI, 0.68–0.7) (Fig. 2). These improvements in AUC values suggest that incorporating aPRS into the models enhances their ability to discriminate between cases and controls for obesity, CAD, and T2D. Following the downsampling process outlined in the methods section, we did not identify any substantial differences in the performance of the model Supplementary Fig. 1.

Additionally, we observed improvements in the Area Under the Precision-Recall Curve (AUPRC) values for all three conditions when aPRS was incorporated into the models. The detailed AUPRC values can be found in Supplementary Fig. 2, which highlights the enhanced precision-recall balance achieved by including aPRS in the models. This further supports the conclusion that aPRS is a valuable addition to the models for predicting the risk of obesity, CAD, and T2D. AUROC and AUPRC values are provided in Supplementary Fig. 2. The models performance in EUR and SAS showed similar trends for AUROC Supplementary Fig. 3 and AUPRC Supplementary Fig. 4.

### Disease association with aPRS categorization in South Asians

Our investigation into the performance of aPRS on SAS individuals revealed an increasing in the risk of developing coronary artery disease (CAD) based on aPRS categorization. Individuals with a low aPRS demonstrated significantly reduced odds of developing CAD, with an odds ratio (OR) of 0.56 (95% CI: 0.45–0.7), indicating a lower risk than the reference group. Conversely, those with a high aPRS exhibited an elevated CAD risk, with an OR of 1.72 (95% CI: 1.44–2.05).

**Fig. 3:**
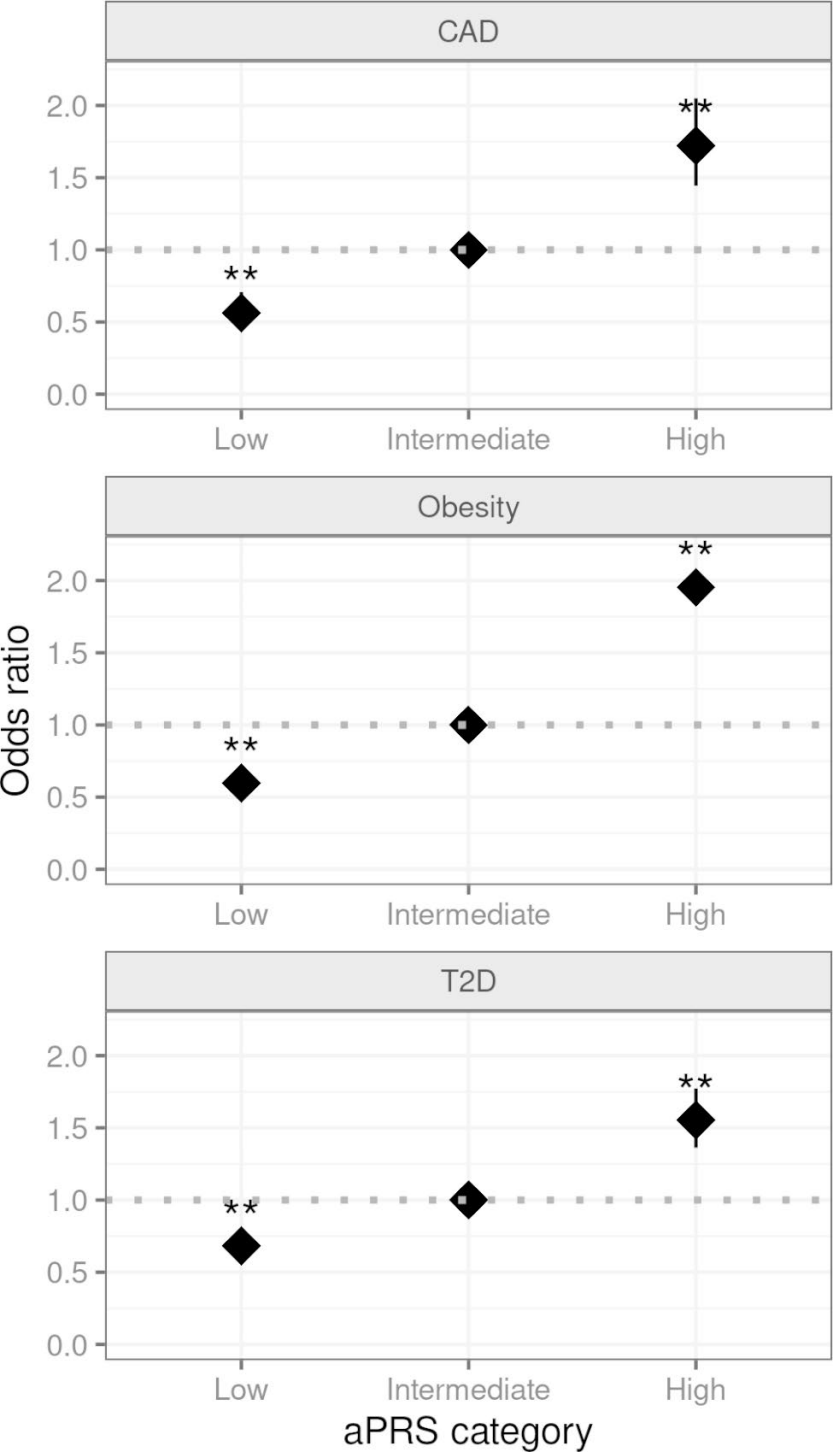
Odds ratio for CAD, and T2D based on the categorization of based on the adjusted polygenic risk scores (aPRS) percentile in the South Asian (SAS) population of the UK Biobank. Coronary artery disease (CAD), type 2 diabetes (T2D). If a p-value is less than 0.01, it is flagged with two stars (**)

Similarly, in the SAS population, the association between aPRS categorization and obesity risk showed similar results. Individuals in the high aPRS group had an OR of 1.95 (95% CI = 1.71–2.23) compared to those in the intermediate aPRS group. Regarding type 2 diabetes (T2D), the high aPRS group in the SAS population had an OR of 1.55 (95% CI, 1.36–1.77) (Fig. 3). While comparing with the EUR individuals a similar trend has been observed Supplementary Fig. 5.

### Association of CAD and T2D with family history and aPRS

**Fig. 4:**
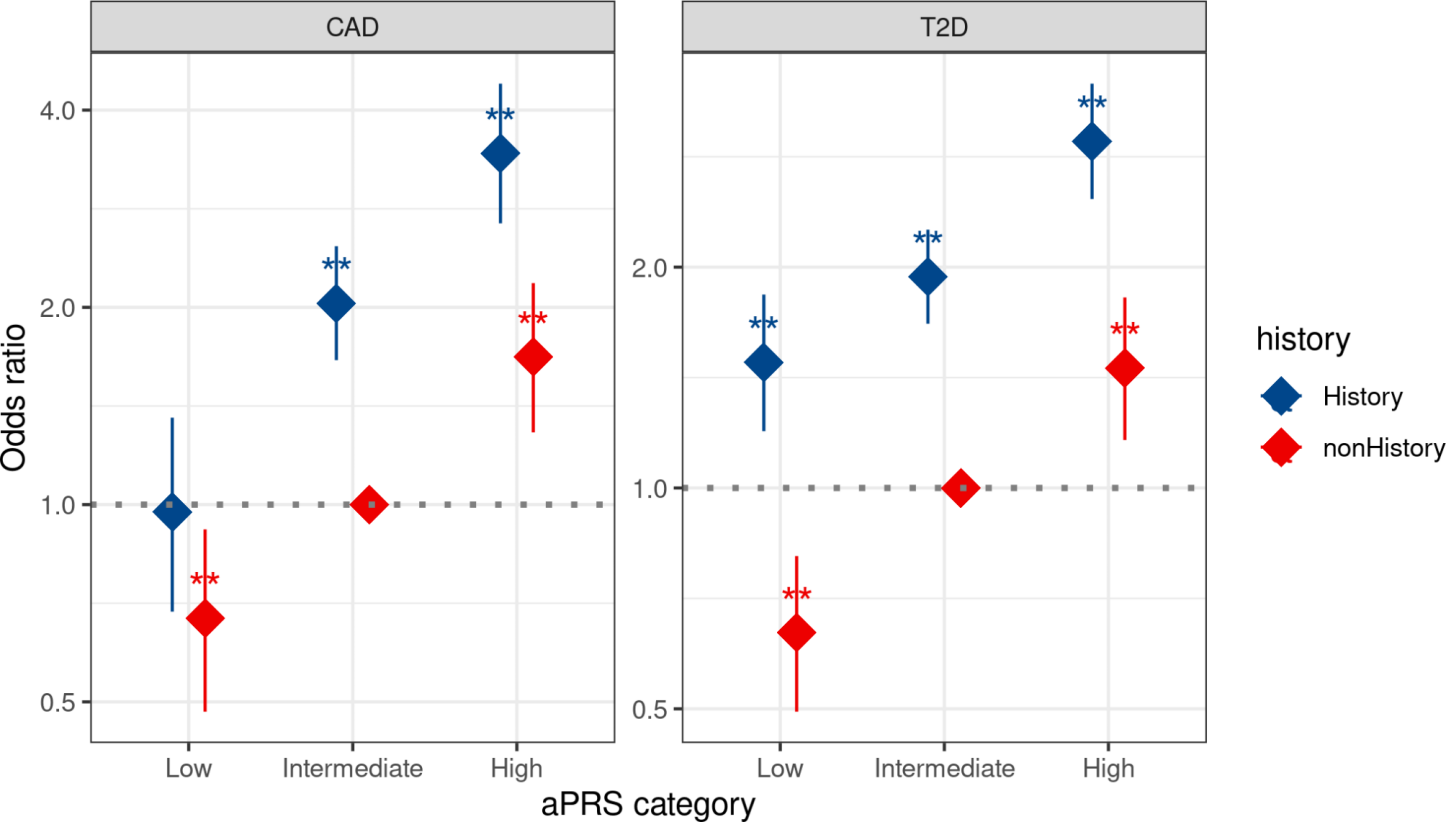
Odds ratio for CAD, and T2D based on the categorization of based on the adjusted polygenic risk scores (aPRS) percentile and family history (FH) status in the South Asian (SAS) and European (EUR) population of the UK Biobank. Coronary artery disease (CAD), type 2 diabetes (T2D), and adjusted polygenic risk scores (aPRS). If a p-value is less than 0.01, it is flagged with two stars (**)

Individuals with a positive FH and high aPRS showed a higher risk of developing CAD than those with no FH and intermediate aPRS (Fig. 4). In SAS, those with both positive FH and high aPRS had a more than three-fold increased chance of developing CAD compared to those with intermediate aPRS and no FH, while individuals with a low aPRS and no FH showed a reduced chance of developing CAD with an OR of 0.63 (95%,0.48–0.91). No significant interaction was observed between FH status and PRS p = 0.11, respectively) (Fig. 4). Notably, in both SAS and EUR, individuals with negative FH and high aPRS had comparable risks of developing CAD as those with positive FH and intermediate aPRS (2-fold risk) Supplementary Fig. 6. The same trend was also shown in T2D.

### Cox-proportional hazard analysis

For the cox-proportional hazard, the Schoenfeld tests conducted on each covariate and the global test do not yield statistically significant results. Consequently, we can reasonably conclude that the assumption of proportional hazards is not violated Supplementary Table 1.

**Fig. 5:**
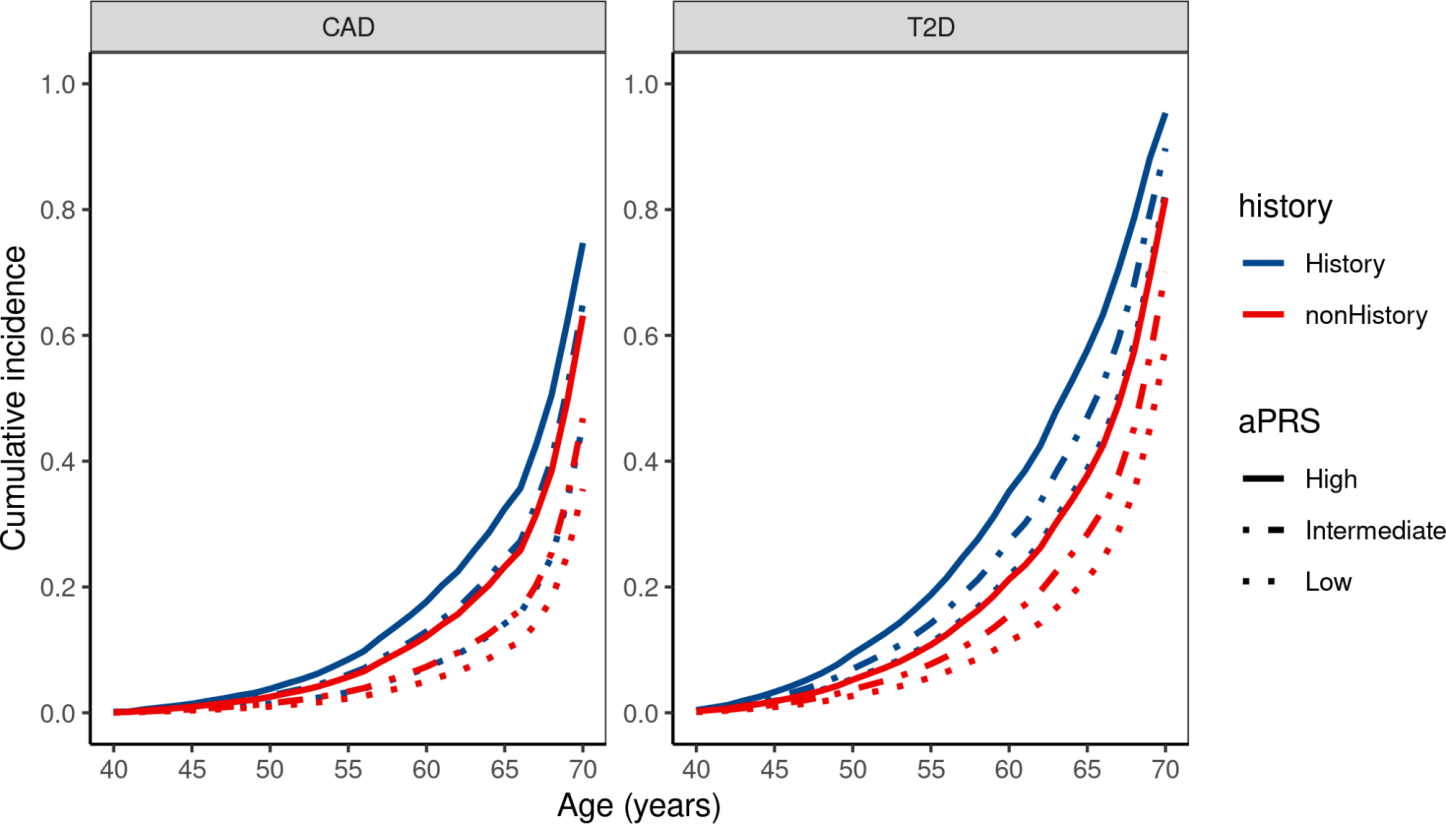
Cumulative incidence of CAD, T2D, and obesity based on the categorization of based on the adjusted polygenic risk scores (aPRS) percentile and family history (FH) status in the South Asian (SAS) population of the UK Biobank. Coronary artery disease (CAD), type 2 diabetes (T2D).

The cumulative CAD incidence among SAS with positive FH increased from 46% with low aPRS to 75% with high aPRS by age 70 (Fig. 5). Notably, SAS individuals with an intermediate aPRS and a positive FH had a cumulative CAD incidence by age 70 (65%) comparable to those with a high aPRS and a negative FH (63%). The cumulative incidence of T2D among SAS individuals ranges from 58% with a negative FH and low aPRS to 95% with a positive FH and high aPRS (Fig. 5). The cumulative incidence of T2D among individuals with high aPRS of SAS ancestry (95%) was higher than EUR individuals (70%) in the corresponding aPRS groups Supplementary Fig. 7.

## Discussion

Extending the previous studies, we aimed to assess the performance of EUR-derived PRSs in the SAS population and explore the relationship between PRS and FH in contributing to the burden of CAD, T2D, and obesity. The results of this study, utilizing UK Biobank data, suggest that an aPRS derived from a large-scale GWAS of cardiometabolic diseases in individuals of European (EUR) ancestry could potentially identify those with an elevated risk of disease predisposition in the South Asian (SAS) population, albeit with a reduced performance observed in the EUR ancestry group. Additionally, the aPRS may identify SAS individuals with increased risk for T2D and CAD independent of their FH. Among high aPRS individuals with positive FH, we noticed an increased cumulative incidence in individuals of SAS ancestry compared to EUR individuals stratified by PRS (Fig. 5).

It has been shown that the UKB is a valuable resource for evaluating the utility of PRS, as it provides both phenotypic and genotypic data [29]. While most UKB participants have EUR ancestry, the dataset involves more than 20,000 participants of self-reported non-EUR.

However, a major challenge with using PRS in clinical settings is that the distribution of genetic variants can vary widely among different ethnic populations [8]. This can result in inaccurate disease risk predictions and hinder the validation of PRS in diverse populations (see Fig. 1). The observed dissimilarity between the distributions for EURs and SASs highlights the need to adjust for the correct ancestral background to accurately assign an individual to their respective percentile within the reference distribution.

We have used population structure adjustment [20] to address this issue, accounting for the genetic differences between different populations when calculating PRS. By adjusting for population structure, we minimized the impact of genetic variability on the accuracy of PRS predictions and facilitate the validation of PRS in diverse populations.

The generalizability of the study’s findings is subject to limitations stemming from several factors. The study participants were recruited exclusively within the UK, including individuals of EUR and SAS ancestry. Thus, healthcare access and non-genetic risk factors may be more comparable among these ethnic groups as they would be expected using two cohorts recruited in EUR and SAS separately. Nevertheless, it is important to acknowledge that socioeconomic determinants, lifestyle choices, and health disparities may differ across various ethnic groups, even living in the same region. Although certain risk variants are likely specific to certain populations, the findings indicating the similar performance of the PRS across ancestry groups suggest that non-EUR groups, including SAS, may share some of the identified risk variants found in EUR-based GWAS for cardiometabolic disorders.

The findings of our study reveal that a higher PRS was associated with an increase in obesity, T2D, and CAD cases among individuals of SAS ancestry. However, the performance of the EUR-based PRSs was less effective in the African (AFR) population, suggesting the existence of ancestry-specific differences [30]. Hence, PRSs should be evaluated carefully by ancestry groups to assess their transferability across ancestries and diseases. Whenever possible, PRS should be constructed based on GWAS based on the same ancestry group [31].

PRS derived from EUR GWAS may not be optimal for all diseases in non-EUR populations, but they can still offer some value in risk assessments for specific conditions [32]. Postponing implementation until ancestry-specific GWAS or multi-ancestry meta-analyses become available could unintentionally widen health disparities across various populations. In the meantime, while larger non-European cohorts are being established, our study illustrates that employing an adjusted PRS based on a EUR GWAS population can provide a limited level of risk categorization for metabolic traits in SAS individuals. However, additional validation is required to ascertain its efficacy.

The increasing availability of data from larger and more diverse populations, coupled with technological advancements, has spurred interest in the clinical adoption of PRS. Recent research has demonstrated that combining clinical risk scores with PRS can help identify more people at risk of developing T2D, especially in SAS populations. Our study provides a potential model for laboratories and health systems seeking to utilize a EUR-derived PRS in SAS populations. Additionally, our study contributes to the literature that supports using PRS and FH as complementary measures in assessing inherited disease susceptibility for T2D and CAD [5].

One of the key findings of our study is the potential improvement in risk prediction when combining family history with PRS [33]. Several theoretical bases support this notion. Family history might reflect the presence of rare genetic variants that are not included in PRS as they are typically constructed from common genetic variants. Additionally, family members often share similar environments and lifestyles, which can contribute to disease risk and may be captured by family history. This shared environment can also influence gene-environment interactions, another potential risk factor for disease. Furthermore, the disease penetrance, or the likelihood that an individual carrying a particular genetic variant will manifest the disease, can also be impacted by family history. Integrating PRS and family history can offer a more holistic estimate of disease risk, encompassing additional genetic and environmental factors. However, the degree to which this combination improves risk estimation depends on the disease and populations under study.

## Conclusion

Taken together, our study provides preliminary evidence that EUR-derived PRSs might be useful to identify individuals at high risk of T2D, obesity, and CAD in the SAS populations. With future GWAS recruiting more SAS participants and tailoring the PRSs towards SAS ancestry, the predictive power of PRS is likely to improve further. Further, we explored the importance of considering both polygenic risk and family history in assessing disease risk in clinical practice. Such an integration could potentially improve risk prediction and provide personalized prevention and management strategies for the common non-communicable diseases. Further research is needed to assess the clinical utility and cost-effectiveness of implementing these measures in diverse populations.

## Electronic supplementary material

Below is the link to the electronic supplementary material.

Supplementary Material 1

## Data Availability

Genome-wide genotyping data, exome-sequencing data, and phenotypic data from the UK Biobank are available upon successful project application (http://www.ukbiobank.ac.uk/about-biobank-uk/). Restrictions apply to the availability of these data, which were used under license for the current study (Project ID: 52,446). Please contact the corresponding author for any data related queries. Data are however available from UK Biobank (see https://www.ukbiobank.ac.uk/enable-your-research for the application procedure).

## References

[1] Sollis E, Mosaku A, Abid A, Buniello A, Cerezo M, Gil L et al. The NHGRI-EBI GWAS catalog: knowledgebase and deposition resource. Nucleic Acids Res 2022 Nov 9;51(D1):D977–85. 10.1093/nar/gkac1010 PMC9825413 36350656

[2] Visscher PM, Wray NR, Zhang Q, Sklar P, McCarthy MI, Brown MA, et al. 10 years of GWAS Discovery: Biology, function, and translation. Am J Hum Genet. 2017 Jul;6(1):5–22. 10.1016/j.ajhg.2017.06.005 PMC5501872 28686856

[3] Khera AV, Chaffin M, Aragam KG, Haas ME, Roselli C, Choi SH, et al. Genome-wide polygenic scores for common diseases identify individuals with risk equivalent to monogenic mutations. Nat Genet. 2018 Sep;50(9):1219–24. 10.1038/s41588-018-0183-z PMC6128408 30104762

[4] Fahed AC, Wang M, Homburger JR, Patel AP, Bick AG, Neben CL et al. Polygenic background modifies penetrance of monogenic variants for tier 1 genomic conditions. Nat Commun 2020 Aug 20;11(1):3635. 10.1038/s41467-020-17374-3 PMC7441381 32820175

[5] Hassanin E, May P, Aldisi R, Spier I, Forstner AJ, Nöthen MM et al. Breast and prostate cancer risk: The interplay of polygenic risk, rare pathogenic germline variants, and family history. Genetics in Medicine. 2022 Mar 1;24(3):576–85. 10.1016/j.gim.2021.11.009 34906469

[6] Hassanin E, Spier I, Bobbili DR, Aldisi R, Klinkhammer H, David F, et al. Clinically relevant combined effect of polygenic background, rare pathogenic germline variants, and family history on colorectal cancer incidence. BMC Med Genom. 2023 Mar;5(1):42. 10.1186/s12920-023-01469-z PMC9987090 36872334

[7] Aldisi R, Hassanin E, Sivalingam S, Buness A, Klinkhammer H, Mayr A et al. GenRisk: a tool for comprehensive genetic risk modeling. Bioinformatics. 2022 May 1;38(9):2651–3. 10.1093/bioinformatics/btac152 PMC9048672 35266528

[8] Privé F, Aschard H, Carmi S, Folkersen L, Hoggart C, O’Reilly PF et al. Portability of 245 polygenic scores when derived from the UK Biobank and applied to 9 ancestry groups from the same cohort. Am J Hum Genet 2022 Jan 6;109(1):12–23. 10.1016/j.ajhg.2021.11.008 PMC8764121 34995502

[9] Li Y, Willer C, Sanna S, Abecasis G (2009). Genotype imputation. Annu Rev Genomics Hum Genet.

[10] Wang M, Menon R, Mishra S, Patel AP, Chaffin M, Tanneeru D et al. Developing Genome-wide Polygenic Risk Scores for Coronary Artery Disease in South Asians. J Am Coll Cardiol. 2020 Aug 11;76(6):703–14. 10.1016/j.jacc.2020.06.024 PMC7592606 32762905

[11] Fatumo S, Chikowore T, Choudhury A, Ayub M, Martin AR, Kuchenbaecker K. A roadmap to increase diversity in genomic studies. Nat Med. 2022 Feb;28(2):243–50. 10.1038/s41591-021-01672-4 PMC7614889 35145307

[12] Fritsche LG, Ma Y, Zhang D, Salvatore M, Lee S, Zhou X et al. On cross-ancestry cancer polygenic risk scores. PLoS Genet 2021 Sep 16;17(9):e1009670. 10.1371/journal.pgen.1009670 PMC8445431 34529658

[13] Hodgson S, Huang QQ, Sallah N, Genes & Health Research Team, Griffiths CJ, Newman WG, et al. Integrating polygenic risk scores in the prediction of type 2 diabetes risk and subtypes in british Pakistanis and Bangladeshis: a population-based cohort study. PLoS Med. 2022 May;19(5):e1003981. 10.1371/journal.pmed.1003981 PMC9119501 35587468

[14] Ho WK, Tan MM, Mavaddat N, Tai MC, Mariapun S, Li J et al. European polygenic risk score for prediction of breast cancer shows similar performance in asian women. Nat Commun 2020 Jul 31;11(1):3833. 10.1038/s41467-020-17680-w PMC7395776 32737321

[15] Duncan L, Shen H, Gelaye B, Meijsen J, Ressler K, Feldman M et al. Analysis of polygenic risk score usage and performance in diverse human populations. Nat Commun 2019 Jul 25;10:3328. 10.1038/s41467-019-11112-0 PMC6658471 31346163

[16] Generalizing polygenic risk. scores from Europeans to Hispanics/Latinos - Grinde – 2019 - Genetic Epidemiology - Wiley Online Library [Internet]. [cited 2022 Sep 29]. Available from: https://onlinelibrary.wiley.com/doi/10.1002/gepi.22166 10.1002/gepi.22166 PMC6330129 30368908

[17] Yang S, Zhou X. Accurate and scalable construction of polygenic scores in large Biobank Data Sets. Am J Hum Genet 2020 May 7;106(5):679–93. 10.1016/j.ajhg.2020.03.013 PMC7212266 32330416

[18] Curtis D. Polygenic risk score for schizophrenia is more strongly associated with ancestry than with schizophrenia. Psychiatr Genet. 2018 Oct;28(5):85–9. 10.1097/YPG.0000000000000206 30160659

[19] Wang M, Menon R, Mishra S, Patel AP, Chaffin M, Tanneeru D et al. Validation of a Genome-Wide Polygenic Score for Coronary Artery Disease in South Asians. Journal of the American College of Cardiology. 2020 Aug 11;76(6):703–14. 10.1016/j.jacc.2020.06.024 PMC7592606 32762905

[20] Hao L, Kraft P, Berriz GF, Hynes ED, Koch C, Korategere V, Kumar P, et al. Development of a clinical polygenic risk score assay and reporting workflow. Nat Med. 2022 May;28(5):1006–13. 10.1038/s41591-022-01767-6 PMC9117136 35437332

[21] Barnett AH, Dixon AN, Bellary S, Hanif MW, O’Hare JP, Raymond NT et al. Type 2 diabetes and cardiovascular risk in the UK south Asian community. Diabetologia. 2006 Oct 1;49(10):2234–46. 10.1007/s00125-006-0325-1 16847701

[22] Mars N, Lindbohm JV, della Briotta Parolo P, Widén E, Kaprio J, Palotie A et al. Systematic comparison of family history and polygenic risk across 24 common diseases. The American Journal of Human Genetics. 2022 Dec 1;109(12):2152–62. 10.1016/j.ajhg.2022.10.009 PMC9748261 36347255

[23] Hassanin E, Spier I, Bobbili DR, Aldisi R, Klinkhammer H, David F et al. Clinically relevant combined effect of polygenic background, rare pathogenic germline variants, and family history on colorectal cancer incidence [Internet]. medRxiv; 2022 [cited 2022 Sep 29]. p. 2022.01.20.22269585. Available from: https://www.medrxiv.org/content/10.1101/2022.01.20.22269585v1 10.1186/s12920-023-01469-z PMC9987090 36872334

[24] Hassanin E, May P, Aldisi R, Krawitz P, Maj C, Bobbili DR. Assessing the role of polygenic background on the penetrance of monogenic forms in Parkinson’s disease [Internet]. medRxiv; 2021 [cited 2022 Oct 6]. p. 2021.06.06.21253270. Available from: https://www.medrxiv.org/content/10.1101/2021.06.06.21253270v1

[25] Lambert SA, Gil L, Jupp S, Ritchie SC, Xu Y, Buniello A, et al. The polygenic score catalog as an open database for reproducibility and systematic evaluation. Nat Genet. 2021 Apr;53(4):420–5. 10.1038/s41588-021-00783-5 PMC11165303 33692568

[26] Bycroft C, Freeman C, Petkova D, Band G, Elliott LT, Sharp K, et al. The UK Biobank resource with deep phenotyping and genomic data. Nature. 2018 Oct;562(7726):203–9. 10.1038/s41586-018-0579-z PMC6786975 30305743

[27] Khera AV, Chaffin M, Wade KH, Zahid S, Brancale J, Xia R et al. Polygenic prediction of weight and obesity trajectories from birth to adulthood. Cell 2019 Apr 18;177(3):587–596e9. 10.1016/j.cell.2019.03.028 PMC6661115 31002795

[28] Choi SW, O’Reilly PF. PRSice-2: Polygenic Risk Score software for biobank-scale data. Gigascience. 2019 Jul 1;8(7):giz082. 10.1093/gigascience/giz082 PMC6629542 31307061

[29] Conroy MC, Lacey B, Bešević J, Omiyale W, Feng Q, Effingham M, et al. UK Biobank: a globally important resource for cancer research. Br J Cancer. 2023 Feb;128(4):519–27. 10.1038/s41416-022-02053-5 PMC9938115 36402876

[30] Ekoru K, Adeyemo AA, Chen G, Doumatey AP, Zhou J, Bentley AR, et al. Genetic risk scores for cardiometabolic traits in sub-saharan african populations. Int J Epidemiol. 2021 Mar;17(4):1283–96. 10.1093/ije/dyab046 PMC8407873 33729508

[31] Graham SE, Clarke SL, Wu KHH, Kanoni S, Zajac GJM, Ramdas S, et al. The power of genetic diversity in genome-wide association studies of lipids. Nature. 2021 Dec;600(7890):675–9. 10.1038/s41586-021-04064-3 PMC8730582 34887591

[32] Huang QQ, Sallah N, Dunca D, Trivedi B, Hunt KA, Hodgson S et al. Transferability of genetic loci and polygenic scores for cardiometabolic traits in british pakistani and bangladeshi individuals. Nat Commun 2022 Aug 9;13(1):4664. 10.1038/s41467-022-32095-5 PMC9363492 35945198

[33] Hujoel MLA, Loh PR, Neale BM, Price AL. Incorporating family history of disease improves polygenic risk scores in diverse populations. Cell Genom. 2022 Jul;13(7):100152. 10.1016/j.xgen.2022.100152 PMC9351615 35935918

