# Supplementary material for: Assessing the performance of European-derived cardiometabolic polygenic risk scores in South-Asians and their interplay with family history
Source: BMC Med Genomics. 2023 Jul 12;16:164. doi: 10.1186/s12920-023-01598-5 (PMC10339617; doi:10.1186/s12920-023-01598-5)
Supplement: Supplementary file 1 — Supplementary Material 1 [file 12920_2023_1598_MOESM1_ESM.docx]

**Assessing the performance of European-derived cardiometabolic polygenic risk scores in South-Asians and their interplay with family history.**

Emadeldin Hassanin, Carlo Maj, [Hannah Klinkhammer](https://bmcmedgenomics.biomedcentral.com/articles/10.1186/s12920-023-01469-z#auth-Hannah-Klinkhammer), Peter Krawitz, Patrick May, Dheeraj Reddy Bobbil

***
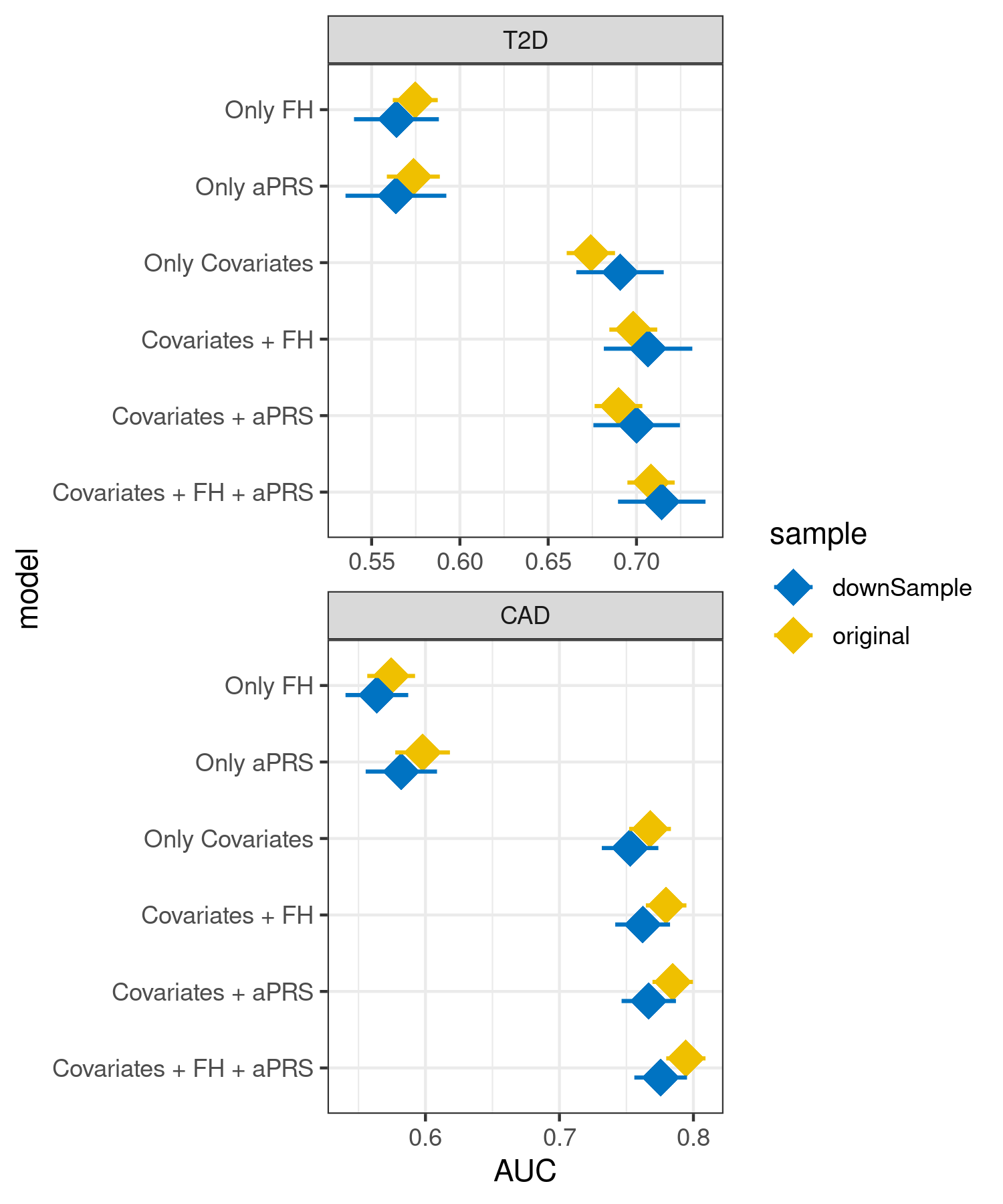
***

***Supplementary Figure 1: Comparison of different models and their corresponding ROC-AUCs before and after down-sampling in the South Asian (SAS) of the UK Biobank.***

*Ancestry adjusted PRS (aPRS), First degree family history (FH) and covariates (age, sex, first four principal components). Coronary artery disease (CAD), type 2 diabetes (T2D).*

***
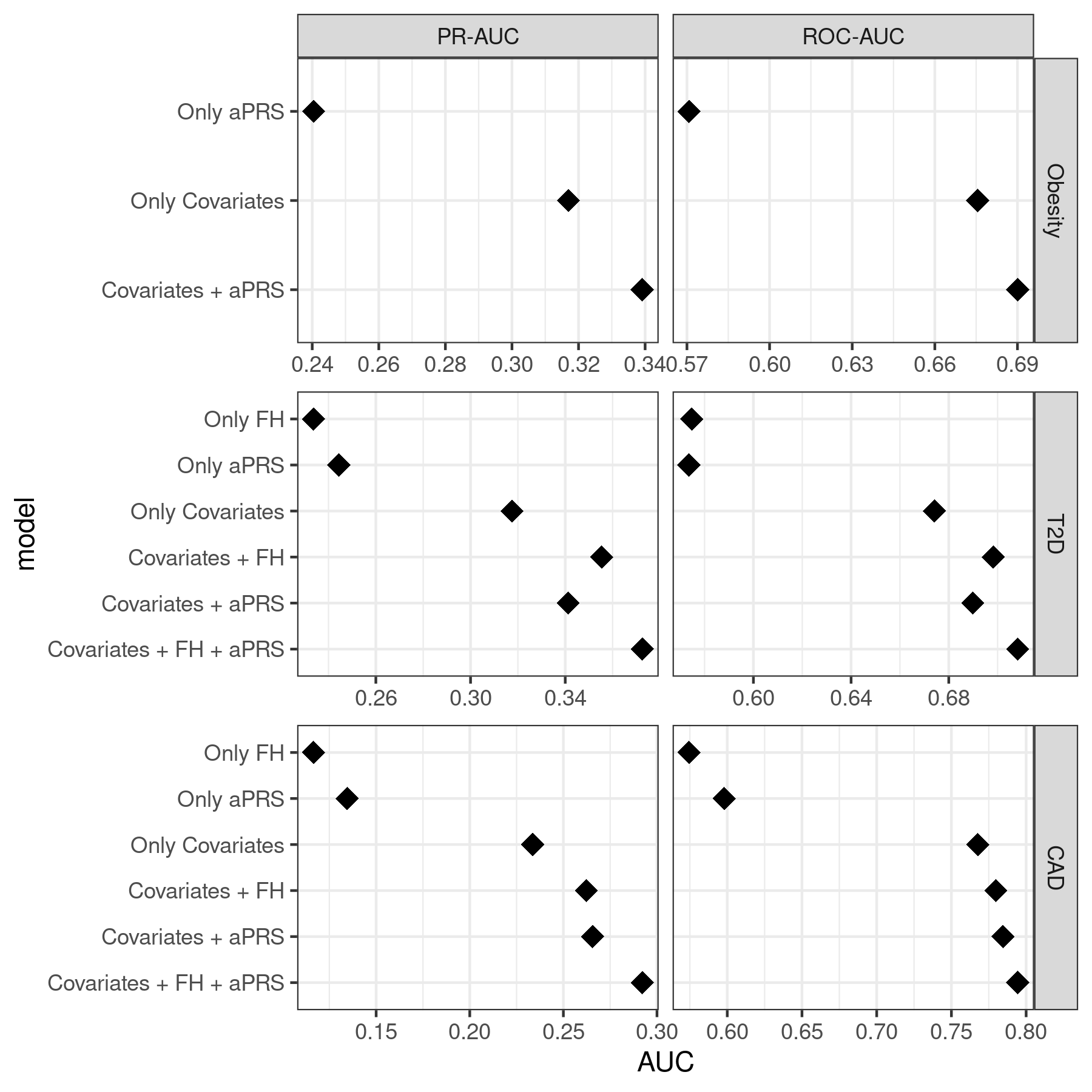
Supplementary Figure 2: Comparison of different models and their corresponding ROC-AUCs and PR-AUCs in the South Asian population of the UK Biobank (SAS).***

*Ancestry adjusted PRS (aPRS), First degree family history (FH) and covariates (age, sex, first four principal components). Coronary artery disease (CAD), type 2 diabetes (T2D).*

*
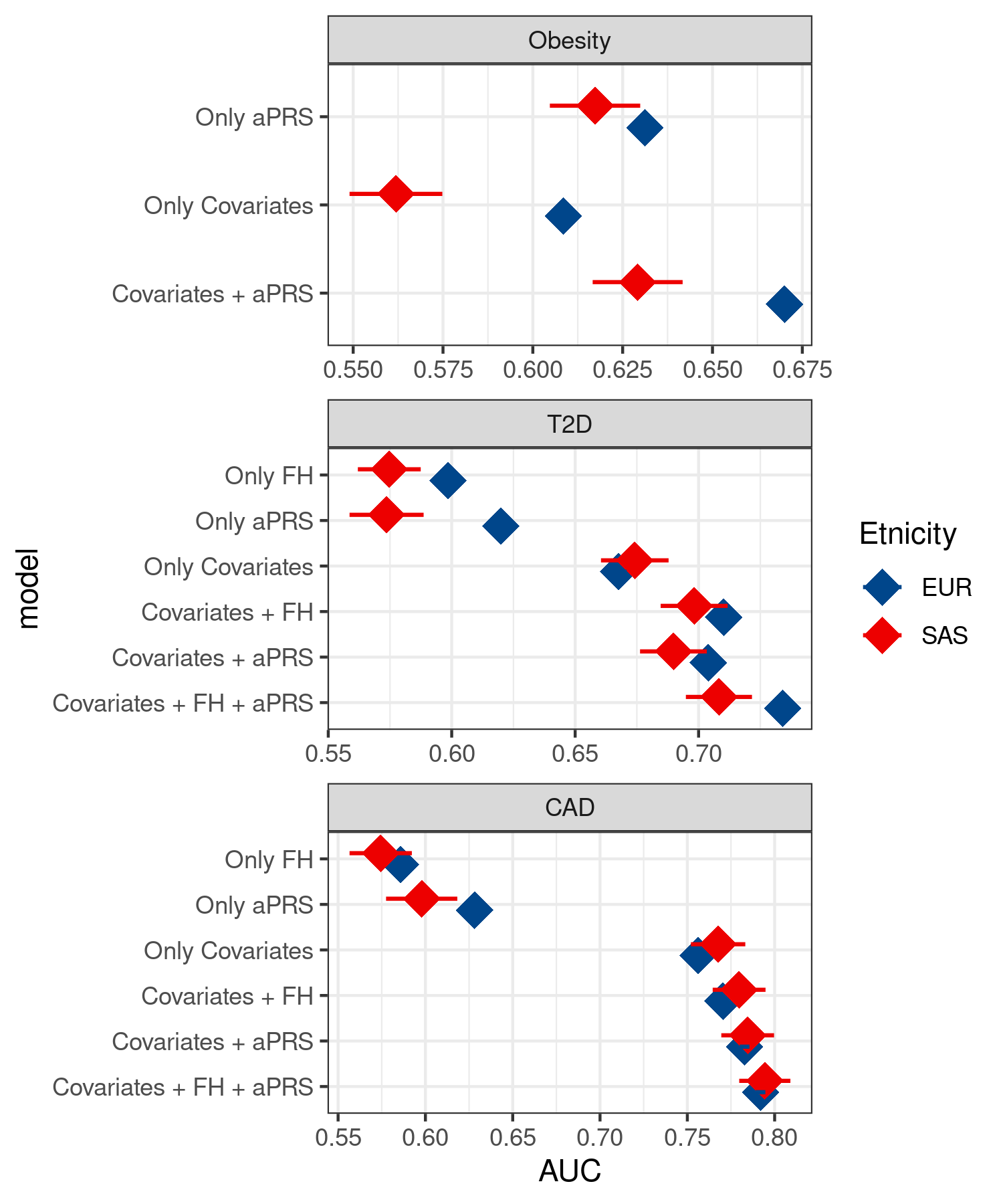
*

***Supplementary Figure 3: Comparison of different models and their corresponding AUCs in the South Asian (SAS) and European (EUR) population of the UK Biobank.***

*Ancestry adjusted PRS (aPRS), First degree family history (FH) and covariates (age, sex, first four principal components). Coronary artery disease (CAD), type 2 diabetes (T2D).*

***
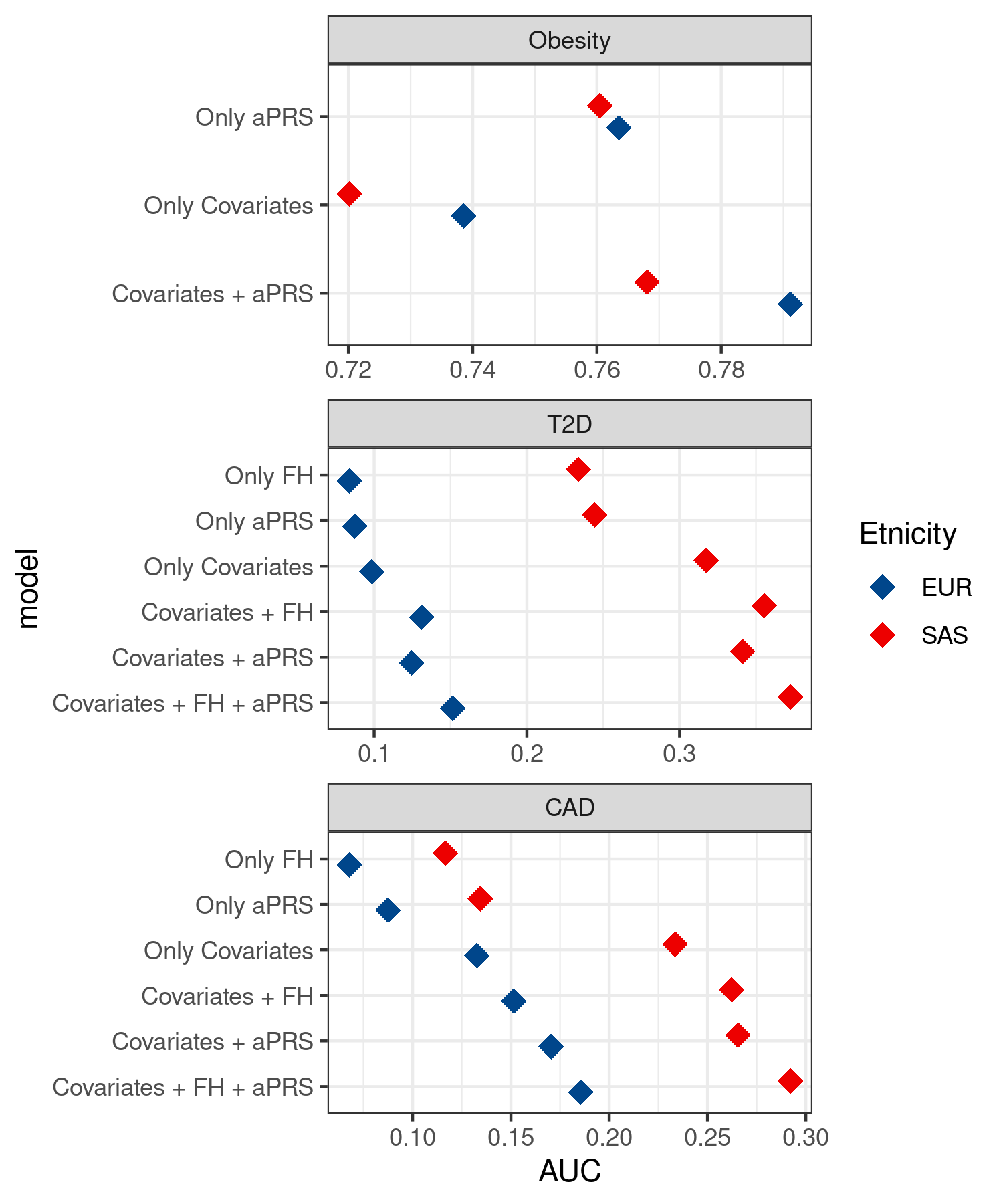
***

***Supplementary Figure 4: Comparison of different models and their corresponding Precision-recall AUCs (PR-AUC) in the South Asian (SAS) and European (EUR) population of the UK Biobank.***

*Ancestry adjusted PRS (aPRS), First degree family history (FH) and covariates (age, sex, first four principal components). Coronary artery disease (CAD), type 2 diabetes (T2D).*

***
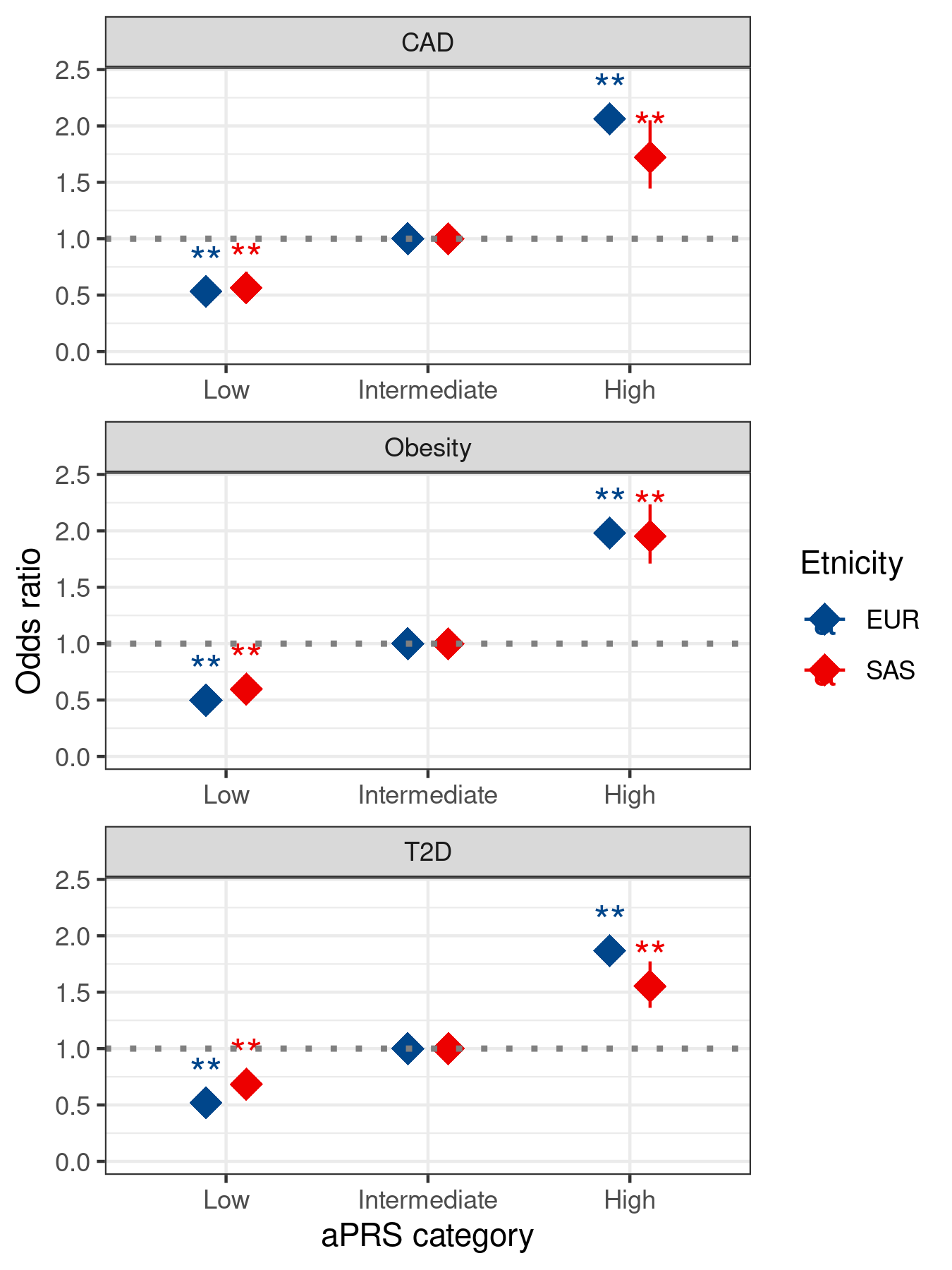
Supplementary Figure 5: Odds ratio for CAD, obesity and T2D based on the adjusted polygenic risk scores (aPRS) categorization in the South Asian (SAS) and European (EUR) population of the UK Biobank.***

*Coronary artery disease (CAD), type 2 diabetes (T2D), and adjusted polygenic risk scores (aPRS). If a p-value is less than 0.01, it is flagged with two stars (**).*

***
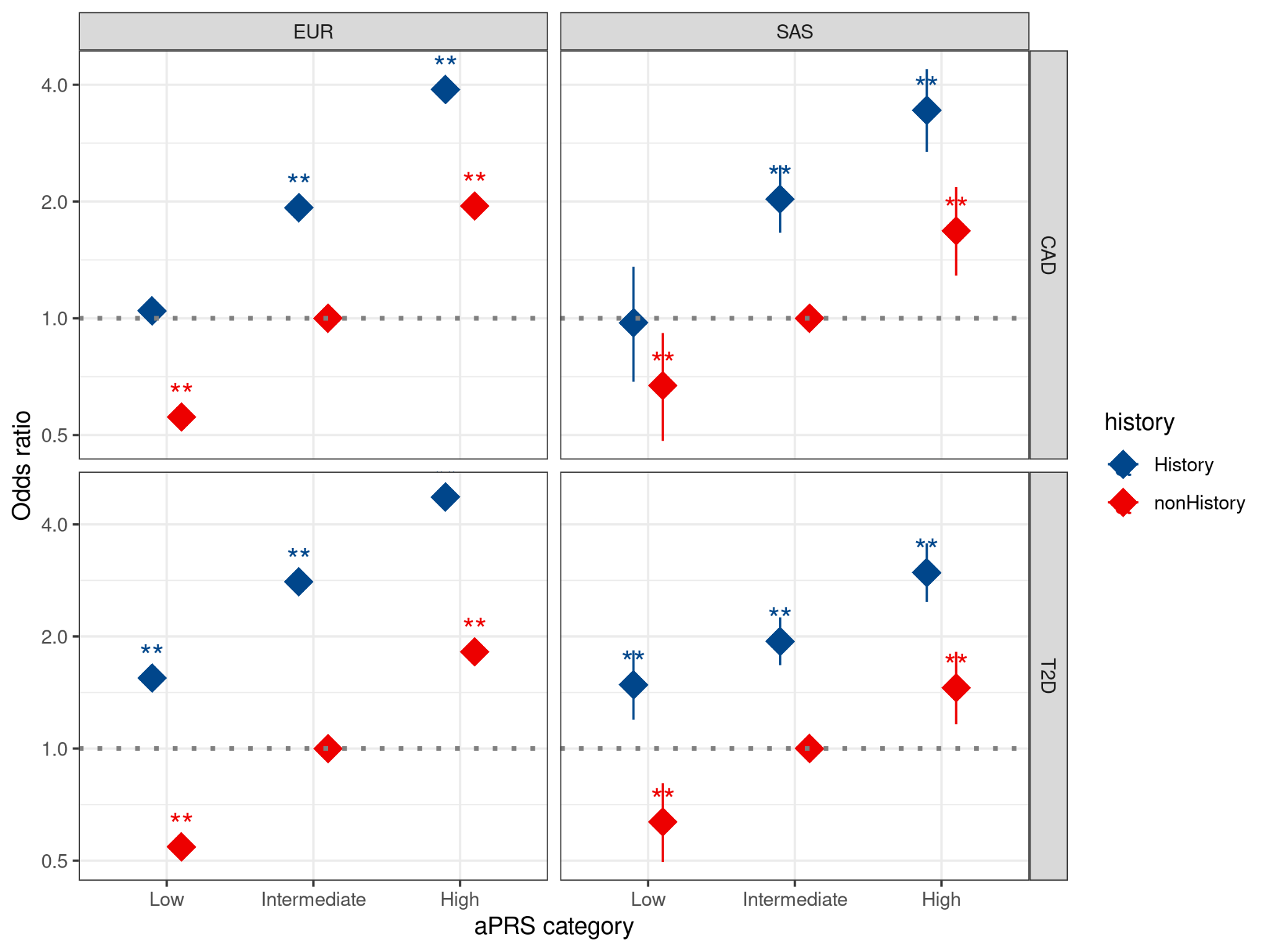
***

***Supplementary Figure 6: Odds ratio for CAD, and T2D based on the categorization of the adjusted polygenic risk scores (aPRS) percentile and family history (FH) status in the South Asian (SAS) and European (EUR) population of the UK Biobank.***

*Coronary artery disease (CAD), type 2 diabetes (T2D). If a p-value is less than 0.01, it is flagged with two stars (**).*

***
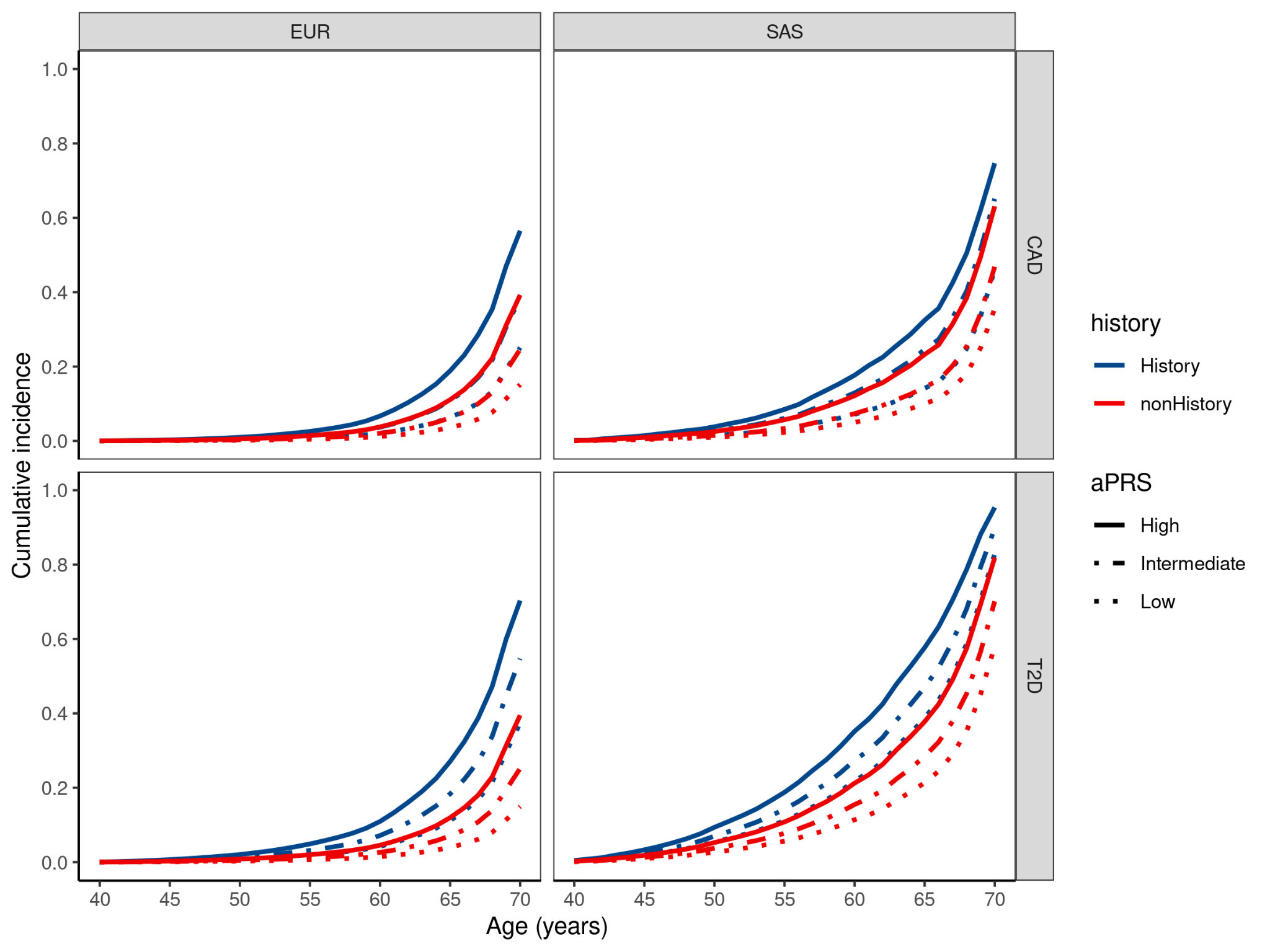
***

***Supplementary Figure 7: Cumulative incidence of CAD, and T2D based on the categorization of the adjusted polygenic risk scores (aPRS) percentile and family history (FH) status in the South Asian (SAS) and European (EUR) population of the UK Biobank.***

*Coronary artery disease (CAD), type 2 diabetes (T2D).*

| **Coronary artery disease (CAD)** | | | |
| --- | --- | --- | --- |
| Model | rho | chisq | p |
| cad_history_ntile | -0.0143 | 1.440 | 0.920 |
| sex | -0.0005 | 0.190 | 0.663 |
| PC1 | 0.0171 | 1.118 | 0.290 |
| PC2 | -0.0058 | 0.833 | 0.361 |
| PC3 | 0.0182 | 5.049 | 0.025 |
| PC4 | -0.0106 | 4.674 | 0.031 |
| GLOBAL | 0.0210 | 11.575 | 0.315 |
| **Type 2 Diabetes (T2D)** | | | |
| Model | rho | chisq | p |
| T2D_history_ntile | -0.0104 | 1.682 | 0.89 |
| sex | -0.0126 | 0.585 | 0.44 |
| PC1 | -0.0096 | 0.115 | 0.74 |
| PC2 | -0.0303 | 0.103 | 0.75 |
| PC3 | -0.0039 | 1.428 | 0.23 |
| PC4 | 0.0186 | 1.541 | 0.21 |
| GLOBAL | 0.0003 | 13.605 | 0.19 |

***Supplementary Table 1: Proportional hazard assumption for all the variables used in the model for both Coronary artery disease (CAD), Type 2 Diabetes (T2D).***

*CAD_history_ntile and T2D_history_ntile represent the variable derived by combining the category of aPRS (Low, Intermediate and high) and Family History (FH) (Positive or Negative) for CAD and T2D respectively.*
